# Ecofriendly synthesis and characterization of Ni^2+^ codoped silica magnesium zirconium copper nanoceramics for wastewater treatment applications

**DOI:** 10.1038/s41598-022-13785-y

**Published:** 2022-06-14

**Authors:** A. M. Mansour, Bahaa A. Hemdan, Amir Elzwawy, Ali B. Abou Hammad, Amany M. El Nahrawy

**Affiliations:** 1 Solid State Physics Department, National Research Centre, Physics Research Institute, El-Bohooth St., Giza, 12622 Egypt; 2 Water Pollution Research Department, Environmental and Climate Change Research Institute, National Research Centre, El-Bohooth St., Giza, 12622 Egypt; 3 Ceramics Department, National Research Centre, El-Bohouth Str., Giza, 12622 Egypt

**Keywords:** Biomaterials, Materials science

## Abstract

This article investigates the effect of Ni^2+^ content on structural (XRD, XPS), morphological (TEM), and magnetic behaviors of silica magnesium zirconium copper nanoceramics calcined at 800 °C. The sol–gel route is followed for the silica magnesium zirconium copper/(0.0–0.7) Ni^2+^ samples preparation. X-ray photoelectron spectroscopy is employed to analyze the chemical states of elements for the samples. The three representative binding energy magnitudes for O, Ni, and Cu reside at 534, 857, and 979 eV, consecutively. The saturation magnetization constricts with the elevation of Ni^2+^ content, while the magnetic hysteresis loop resembles the superparamagnetic attitude. The optical spectra present the possibility of direct and indirect transitions in the prepared nanoceramics. Energy gap (value and type), refractive index, and real and imaginary dielectric constant were extracted. The energy gap approaches 3.75 eV and 3.71 eV for direct and indirect transitions correspondingly with (0.7) Ni^2+^. The antimicrobial and the toxicity performance of all inspected nanocomposites were conducted against pathogenic microbes. The attained results evidenced that SMZC-0.7Ni possesses energetic antimicrobial potential against all targeted microbes. The investigated SMZC-0.7Ni nanocomposite functioned to eradicate frequent waterborne pathogens in wastewater at an appropriate dose (100 mg/L), demonstrating that SMZC can be utilized as a competent disinfectant in the municipal wastewater decontamination process. Inherently, SMZC-0.7Ni can be employed as an excellent nano-weapon against multiple dangerous microorganisms.

## Introduction

Water is amongst the necessities of human civilization and other life forms in the universe, as it is utilized in various daily activities. Industrial development significantly contributes to the creation of sewage, which is often dumped untreated into bodies of water, resulting in pollution ^1^. Due to rising costs of freshwater resources, increasing population, and a range of climatic and environmental challenges, it is estimated that over 1.1 M people have no access to an appropriate water supply ^2^. Likewise, pathogenic microbes encompassing bacteria, viruses, fungi, protozoa, and rickettsia, trigger 80% of the total illnesses in underdeveloped countries, representing a severe hazard to public health and claiming millions of lives each year around the world ^3^.

The management of wastewater could be a dormant solution to pollution reduction; yet, typical treatment procedures are insufficient to eliminate new pollutants and meet stringent water quality criteria entirely. The chlorination process is employed to inactivate pathogenic microorganisms in wastewater treatment plant effluents (WWTPs) before the point of discharge. Chlorination and ultraviolet (UV) irradiation are the most frequently applied disinfecting techniques ^4^. Nevertheless, the difficulties these two approaches encounter, such as the formation of disinfecting by-products (DBPs) following chlorination and the energy consumption and industrial effluent turbidity limits associated with UV irradiation, are creating concerns ^5^. Combining the abovementioned typical oxidizing disinfectants with numerous constituents (e.g., NOMs) in water produces these DBPs. As a result, an alternative disinfection technology that can achieve excellent disinfection effectiveness while being ecofriendly, energy-saving, manageable to perform, and economically viable is still essential ^6^.

Nano-biotechnology (NBT), a fashionable technology, holds many promises in the field of wastewater disinfection. Because of the rapid progress of nanotechnology, there is a growing interest in exploring the antibacterial properties of various nanomaterials (NMs) and using them in water disinfection processes ^7^. Due to their potential to be stimulated under solar sunlight irradiation, these NMs have demonstrated a tremendous promise to be applied as alternatives to conventional disinfectants and to be associated with other existing technologies to promote disinfection performance, such as photo-excitation ^8^.

Recently, much attention has been concerned to modify novel functional inorganic nanoceramics for use in different applications both in daily lives, drug delivery, water treatment, and industrial productions ^9,10^. Mesoporous silicate nanocomposites have attractive properties such as high surface area, good textural, amenable pores, and distinguished various compositions and structures, which give possible applications in sensors, coating, catalysis, drug delivery, adsorption, and optoelectronics ^7,11,12^. Silica-doped with copper, zirconia, and other metal oxides (MgO, CeO_2_, Ag, etc.) are extensively studied due to their physicochemical properties as the higher surface area pursued the applications that allow high reaction rates ^13^.

The magnetic specifications resulted from the investigation of the subjected material as ferromagnetic, ferrimagnetic, or else sheds light on its structure, configuration, grain size, and domains attitude ^14–16^. The incorporation of high thermally stable nanocrystalline as silicate or highly corrosion resistive materials as zirconium and mixing in a composite can yield elevated microstructural/mechanical and magnetic properties.

In this work, due to numerous substantial advantages, the sol–gel route has been successfully used in the development of silica magnesium zirconium copper nanoceramic suited for various applications ^17^. Sol–gel methods are favored for the performance of several technological operations under lower and milder conditions with higher stability ^18–21^. Sol–gel silicate-based nanoceramics have attracted broad interest owing to their excellent structural and stability, low-cost, and environmentally saving, supporting these silicates for an extensive variety of applications ^22,23^. Hence the homogeneity of sol–gel silicate-based nano-scale depends on various aspects, like particle size, shape, dopant type, and the density of the starting particles and the additives ^24^. The controlled magnetic-silicate matrices behavior can serve in a wide range of applications such as magnetic head recording, hyperthermia, water treatment, and drug delivery ^10^.

This work addresses the higher chemical stability through the sol–gel assembly of silica magnesium zirconium copper (SMZC) framework and doped with (0.0–0.7) Ni^2+^ nanoceramics, supported by various metal precursors from the agglomeration during synthesis, producing higher crystalline mesoporous (SMZC), and functionalized structural integrity of the (SMZC/Ni^2+^) nanoceramics. Promoting a stable microstructure, optical and magnetic properties of silica magnesium zirconium copper (SMZC) doped with (0.0–0.7) Ni^2+^ nanoceramics, synthesized by well-ordered sol–gel reactions at 800 °C are achieved herein. Modification of silicate with Cu, Ni, Mg, and Zr produces active centers with large surface area in silicate-based nanoceramics and with good optical, thermal, and magnetic properties, which support their uses in numerous catalysis, sensors, and other intelligent applications. Adding, Investigation of the structural, optical, and magnetic properties of Ni^2+^ Co-doped silica magnesium zirconium copper nanoceramics and their applications for water and wastewater treatments.

## Experimental

### Chemical constituents

Zirconium nitrate (ZrO(NO_3_)_2_·H_2_O, Merck, Germany), tetraethylorthosilicate (TEOS, (C_8_H_20_O_4_Si), Sigma, Germany), nickel, copper, and magnesium nitrates (Ni(NO_3_)_2_·6H_2_O, CuN_2_O_6_ and MgN_2_O_6_, Sigma, Germany), ethyl absolute (C_2_H_6_O, Alpha, USA), nitric acid (HNO_3_, Alpha, USA). All constituents were used as expected without further purification.

### Silica magnesium zirconium copper doped with Ni^2+^ nanoceramics preparation

Silicate-based nanoceramics (SiO_2_/MgO/ZrO_2/_CuO) doped with three concentrations (0.0, 0.3, 0.5, 0.7) of Ni^2+^ nanoceramics were synthesized by the controlled sol–gel process. To increase the hydrolysis rate, TEOS/ethanol was activated at 50 °C for 20 min in a mixture of H_2_O/0.2 M HNO_3_. Zirconium, copper, and magnesium sources were dissolved in ethanol/H_2_O/HNO_3_ (5/20/0.1 mL) before being added to the silica solution under vigorous stirring for 2 h, to yield (SiO_2_/MgO/ZrO_2/_CuO) sol below stirring for 1 h at 70 °C. Next, the (0.0–0.7)Ni^2+^ doped (SMZC) nanoceramics were prepared by tallying the (SMZC) mixture to nickel solution (Ni(NO_3_)_2_·6H_2_O/ethanol/H_2_O/nitric acid) which stirred at 70 °C.

Finally, the resultant pure and Ni^2+^ doped gels were happened by stirring the sols at 100 °C and then calcined the samples at 800 °C in an electric oven, to give SMZC and Ni^2+^ doped nanoceramics before characterization. X-ray photoelectron spectroscopy using a KRATOS-AXIS: (XPS) Ultra-spectrometer fortified with the monochromatic (hm = 1486.71 eV) α—X-ray source. The (CuKα) source, (λ = 0.15406 nm) of X-ray diffraction (XRD: D8-Bruker) Axs—Advance X-ray diffractometer (Bruker Co., Germany) was working for describing manufactured nanoceramic samples. The particle nanosize of the prepared nanoceramics samples was observed by HR-TEM (JEM-model; Jeol, Japan).

### Evaluation of microbial inactivation efficiency

The biocidal performance of four nanocomposites (SMZC-0.0Ni, SMZC-0.3Ni, SMZC-0.5Ni, and SMZC-0.7Ni) was assessed against four different types of microorganisms including, *Escherichia coli O157:H7, Staphylococcus aureus*, *Candida albicans*, and *Aspergillus niger* using agar diffusion and broth dilution assays. The selected microbial strains were cultivated in Trypticase soy agar (TSA) at 37 °C overnight ^25^.

#### Agar diffusion assay

The antimicrobial capabilities of four innovative nanocomposites were investigated using the disc and well diffusion approaches against the microorganisms above. In this experiment, 100 L of each fresh selected microorganisms culture was carefully disseminated on the top layer of Mueller Hinton agar (MHA; Becton Johnston Co., Sparks, MD). Likewise, the autoclaved paper discs were filled with 50 µL of each studied nanocomposites for the disc diffusion assay and deposited into the inoculated agar plates. At the same time, the same volume of each nanocomposite investigated was injected directly into the wells, perforating MHA's profound layer of MHA agar. After 24 h of plate incubation at 37 °C, The clear zones diameters (CZD) formed were estimated in millimeters (mm) ^26^.

#### Inactivation of cell viability

Using the broth dilution assays, the antimicrobial properties performance of each of the evaluated nanocomposites was checked to obtain the minimum inhibitory concentrations (MICs) values. As explained previously, every microbe examined (10^6^ CFU/mL) was undergo to various concentrations of each nanocomposite solution (25–100 mg/L) for varying retention times (5–30 min). The numbers of developed colonies (around 30–300 CFU) were calculated to quantify microbial populations before and after treatment to particular nanocomposites ^27^. The total number of surviving microbial cells was counted using the platelet count viability technique.

#### Estimation of released protein

Protein amounts released from damaged microbial cells were measured using the Coomassie blue assay. A hundred μL of the microbial suspension was inserted into two tubes of 50 mL sterile Typricase soy broth. One contains the effective dose and the other, which is considered a negative control, was lacking tested nanocomposites. All tubes were placed at 37 °C with agitating at 200 rpm in a shaking incubator, and samples were obtained every 2 h for 24 h (n = 12 readings) ^28,29^.

#### Kinetic modeling for the mechanism of action

The pseudo-first-order was applied for estimating the kinetic modeling to appraise Ascertaining the speed of the effect of this effective concentration on microbial species and determining the fastest destroyed species with the concentration mentioned above.

#### Toxicity assay

The ecotoxicity of four aqueous suspension solutions of four nanocomposites was assessed using the luminescent *Vibrio fischeri* measurement technique. The hypothesis behind this test is that when this marine bacterium is subjected to sample matrices, the intensity of light produced by such bacteria increases or reduces relaying of its metabolic activity. To quantify the toxic level of aqueous solutions, the Microtox 500 analyzer was used to perform, with bioluminescence light output estimates taken after 5, 10, and 15 min as a contact time. For each nanocomposite investigated, EC_50_ of bioluminescence inhibition was estimated using the MicrotoxOmni Azur program ^30,31^.

#### Decontamination of wastewater using innovative nanocomposites

Wastewater water samples, which were collected from the clarifier system of the Abu-Rawash wastewater treatment plant, were decontaminated with an appropriate dosage of the investigated CAS antiseptic. Appropriate techniques were applied to estimate the sewage sample's physicochemical features. Waterborne pathogens such as *E. coli* O157*, Salmonella typhimurium, S. aureus,* and *B. subtil*is were checked in water samples before and after decontamination using the diffusion plate approach ^32^. Each disinfectant’s effective dose (200 ppm) was injected into each flask containing 50 mL of sewage water. After dosage exposure at different varying intervals ranging from 0 to 60 min, a suitable portion (100 µL) was taken from the flask and distributed on the surface of the chromomeric agar medium indicated in previously published articles by Hemdan et al.^21^.

## Results and discussion

### XPS analysis

**Figure 1:**
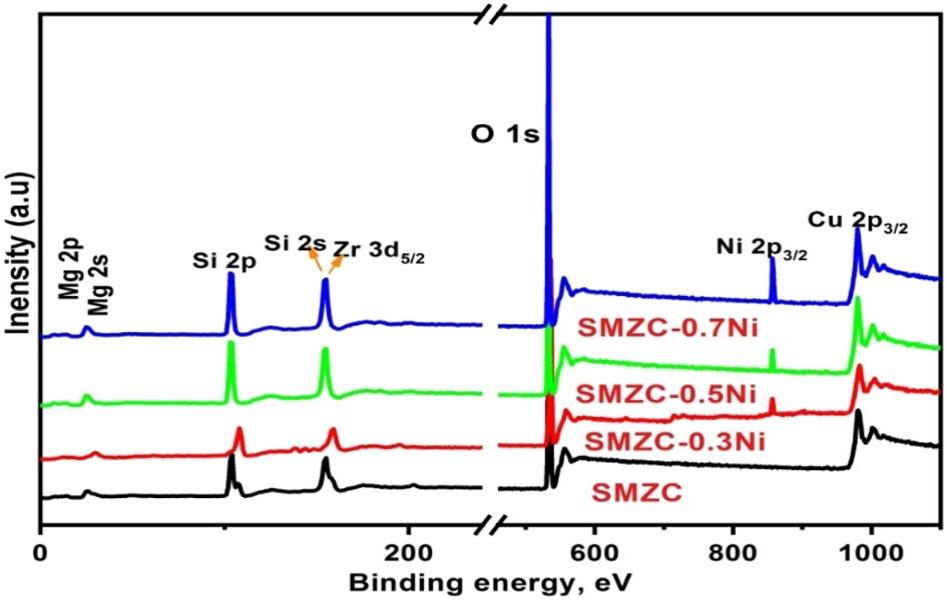
XPS survey of SMZC nanoceramics doped with (0.0–0.7) Ni^2+^, calcined at 800 °C.

To explore the chemical structure of SMZC and (0.0, 0.3, 0.5, 0.7) Ni^2+^-doped nanoceramics, XPS was performed in the binding energy range from 1 to 1100 eV, to obtain their compositional elements (Fig. 1).

The obtained atomic concentrations of the elements for pure and doped samples in the lower binding energy are Mg, Si, and Zr at 12, 24.99, and 154 eV, respectively ^33,34^. The three characteristic binding energy values for O, Ni, and Cu are at 534, 857, and 979 eV, respectively. The higher peak at 534 eV corresponds to the (O 1s) of the Si–O and Si–O–M (M = Mg, Zr, Cu) in SMZC nanoceramic ^34^. The binding energy for Ni^2+^ doped samples is notably higher than that in the pure SMZC nanoceramics, supporting the introduction of Ni^2+^ effects on the local structure of SMZC crystal structure. This result appears to be the ability of XPS rays penetration for the complex SMZC surface and detectable their compositions through the analysis, where Ni concentration increased with decreasing Si in the SMZC matrix. Further, the varying in silicate compositions under the influence of various species (i.e., Mg, Ca, Fe, and Go) using the XPS analysis has been reported ^35,36^.

### X-ray diffraction analysis

**Figure 2:**
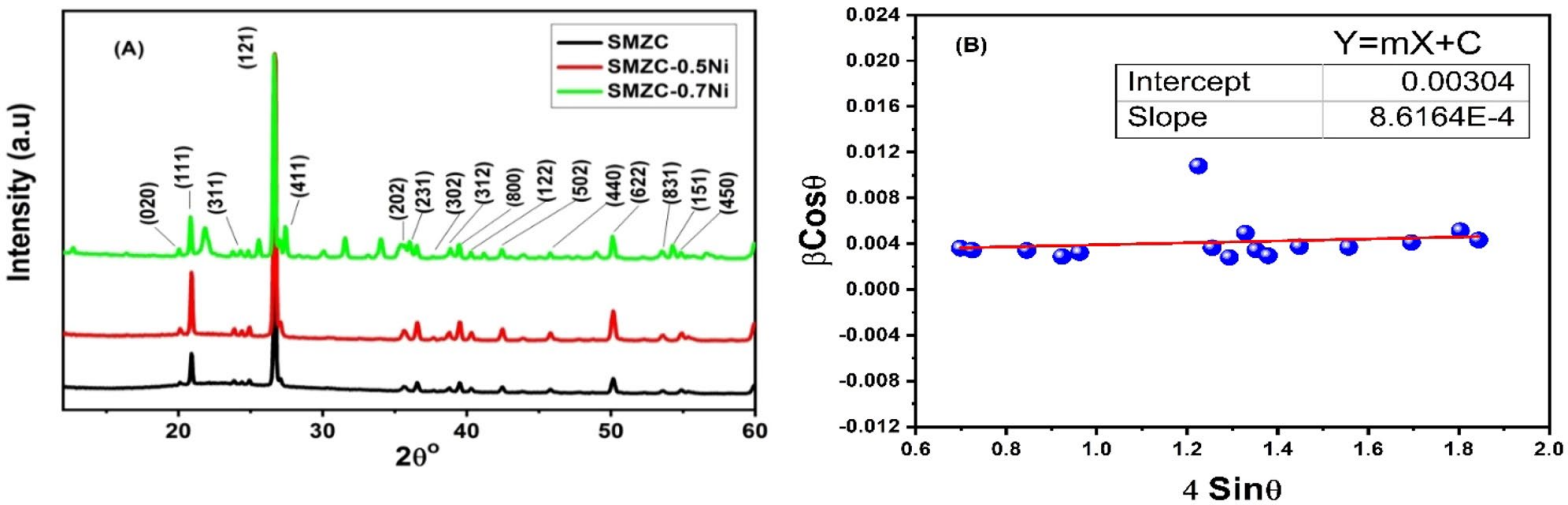
(**A**) XRD spectra of the SMZC/(0, 0.3, 0.5, 0.7) Ni^2+^, calcined at 800 °C, and (**B**) a plot of Williamson-Hall relation of SMZC sample.

Figure 2 shows the diffraction pattern for the prepared SMZC and Ni^2+^ doped nanoceramics, with the indexed diffraction planes, and (B), the Williamson-Hall plot for estimating the crystallite size and the lattice microstrain.

Phase identification for the X-ray diffractogram was performed. Here, we can index the peaks about JCPDS card no. 01-087-0045 for magnesium copper silicate with the chemical formula of Mg (Mg_0.56_, Cu_0.44_) (Si_2_O_6_) and orthorhombic crystal system as stated earlier ^37^. The diffraction line broadening is promoted by the imperfections in the lattice, where defects, vacancies, and dislocations cause lattice strain ^38^. XRD peaks broadening signify the existence of nanocrystals amongst the samples ^39^. Noteworthy to remark the superimposition of the silicate diffraction peaks at 20.87, 26.66, 36.59, 39.51, 40.33, 42.47, 45.89, 50.17, and 55.39 degrees for (100), (101), (110), (012), (111), (200), (201), (112), and (022) planes respectively. This is matching with the JCPDS card no. 03-065-0466 with hexagonal phase as listed previously ^40^. All the crystalline diffraction peaks at about 20.8°, 26.6°, 36.5°, 39.5°, 40.32°, 42.47°, 45.8°, 50.6°, 54.9°, and 55.3° are attributed to the crystalline zirconium silicate according to JCPDS (card no. 81-0589) tetragonal phase ZrSiO4.

Total peaks broadening is a result of the crystallite size and micro-strain contributions expressed as

1 $$\left({\beta }_{total}\right)=\left\{{\beta }_{crystallite}+{\beta }_{strain }\right\}.$$

Therefore,

2 $${\beta }_{total}=\frac{k\lambda }{D \mathrm{cos}\theta }+4\varepsilon \mathrm{tan}\theta ,$$

and finally, by rearranging the equation

3 $$\beta \mathrm{cos}\theta =4\varepsilon \mathrm{sin}\theta +\left[\frac{k\lambda }{D }\right].$$

This is denoted as Williamson-Hall expression for uniform deformation. By plotting a relation on the form of y = mx + c, with *β*cos*θ*, being the y-axis and 4sin*θ* on the x-axis, we can estimate the micro-strain from the linear fit slope, and the crystallite size from the intercept with the y-axis.

The diffraction parameters were calculated and tabulated in Table 1.

**Table 1:** The calculated parameters from the X-ray diffraction pattern, crystallite size, and microstrain compared to the Williamson Hall plot values.

2θ°		(hkl)	d (Å)	θ		β		D (nm)	δ = 1/D^2^ (nm)^–2^ × 10^–4^	ε
Graph	JCPDS card			(°)	(rad)	(°)	(rad) × 10^–3^	Scherrer		Calculated ε = β/4tanθ × 10^–3^
20.12	19.96	(020)	4.45	10.06	0.18	0.21	3.67	38.4	6.78	5.17
20.87	20.28	(111)	4.37	10.43	0.18	0.2	3.49	40.4	6.13	4.74
24.40	24.58	(311)	3.61	12.2	0.21	0.2	3.49	40.6	6.06	4.04
26.69	26.76	(121)	3.33	13.34	0.23	0.17	2.96	48.1	4.32	3.12
27.83	27.83	(411)	3.20	13.91	0.24	0.19	3.32	43.0	5.40	3.35
35.66	35.72	(202)	2.51	17.83	0.31	0.65	1.13	12.8	6.07	8.81
36.58	36.18	(231)	2.48	18.29	0.32	0.22	3.84	38.0	6.92	2.90
37.72	37.43	(302)	2.40	18.86	0.33	0.17	2.96	49.5	4.08	2.17
38.79	38.83	(312)	2.31	19.39	0.34	0.3	5.23	28.1	12.7	3.71
39.51	39.54	(800)	2.27	19.76	0.34	0.21	3.67	40.2	6.20	2.55
40.32	40.31	(122)	2.23	20.16	0.35	0.18	3.14	46.9	4.53	2.14
42.44	42.54	(502)	2.12	21.22	0.37	0.23	4.01	37.1	7.27	2.58
45.81	45.41	(440)	1.99	22.91	0.40	0.23	4.01	37.5	7.10	2.37
50.16	50.40	(622)	1.80	25.08	0.44	0.26	4.53	33.8	8.76	2.42
53.61	53.66	(831)	1.70	26.81	0.47	0.33	5.76	26.9	13.8	2.85
54.91	54.72	(151)	1.67	27.46	0.48	0.28	4.88	32.0	9.76	2.35
55.38	55.47	(450)	1.65	27.69	0.48	0.4	6.98	38.4	6.78	5.17

Crystallite size was estimated using the Scherrer equation and compared to Williamson–the Hall plot. The strain was calculated directly as well as from the slope of the Williamson-Hall graph. The interplanar distance offered by Bragg’s law. The correlation between average crystallite size with HRTEM histogram and Sherrer equation was confirmed.

### TEM

**Figure 3:**
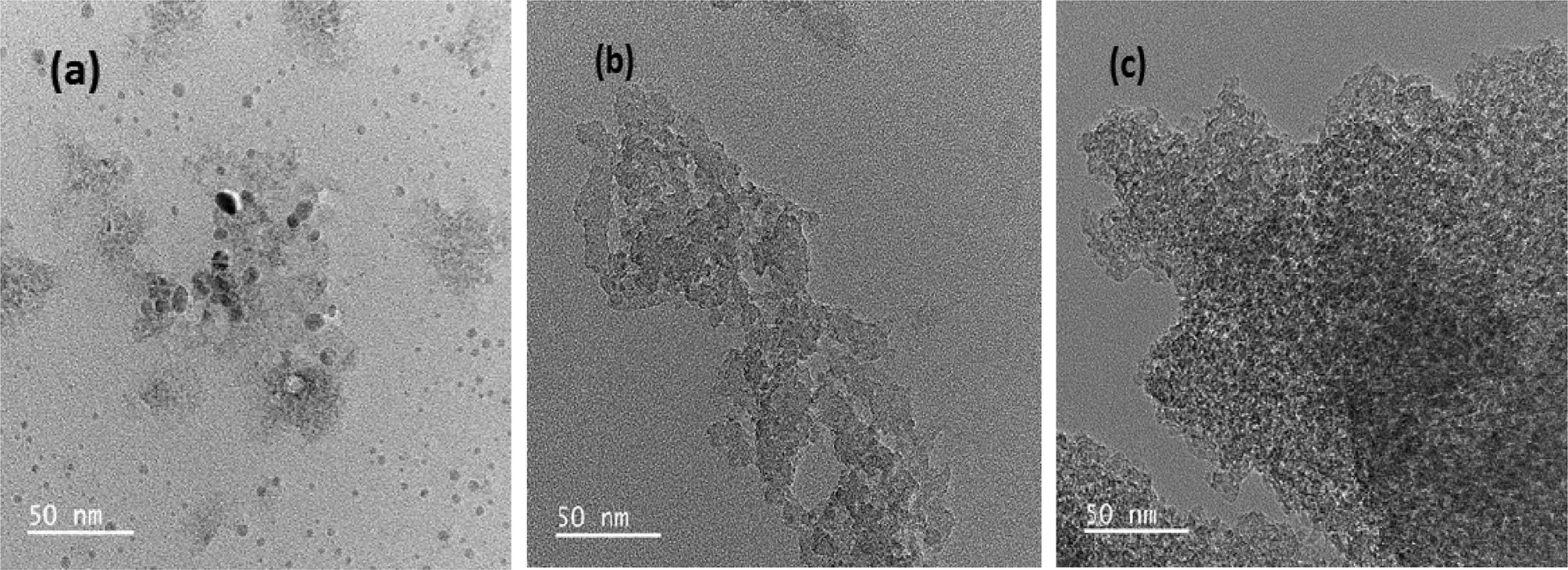
(**a**) Lower magnification TEM image of SMZC nanoceramics doped with (**b**) 0.3, and (**c**) 0.7 Ni^2+^ nanoceramics calcined at 800 °C.

Figure 3 shows lower magnification TEM images of a polycrystalline SMZC doped with (0.3, 0.7) Ni^2+^ nanoceramics. The images show highly ordered SMZC and SMZC-Ni nanoceramics with clustering behavior leading to the well-ordered SMZC nanoceramic: both the fine nanoceramics and nanoclusters appear with slight differences. Lower magnification TEM for the samples showed regular nanoceramics with average nanosized from 7 to 16 nm.

### UV–Vis spectroscopy

Electronic absorption or UV–Vis spectroscopy is unique to the conventional optical techniques for revising the electronic and optical properties of materials, constructed on the capacities of light absorption through the sample ^11,41^. The transmitted light intensity will decrease in case of light absorption by the sample at a certain wavelength. The sample absorption spectrum is explored by plotting the transmitted light intensity against light wavelength. The wavelength range (200–800 nm) is protected by the greatest of the spectrometer. The plain operating of the electronic absorption is based on the assessment of light absorption that occurs as a result of electronic transitions.

The wavelength of light essential for the electronic transition is classically in the ultraviolet and visible sections of the electromagnetic range ^42^.

Using Beer’s law, the absorbance (A) is correlated with the incident light intensity (I_o_), the transmitted light intensity (I), and the concentration of the sample (C) with the path length (L), absorption coefficient (α) and the molar absorptivity (ε) via the next equation^43^

4 $$A=\mathit{log}{I}_{0}/I=\varepsilon LC=\alpha C.$$

Experimentally, (A) can be determined via measuring both (I_o_) and (I). The absorption coefficient is wavelength reliant and the plot of (α) as a function with the λ is the spectrum of concern.

**Figure 4:**
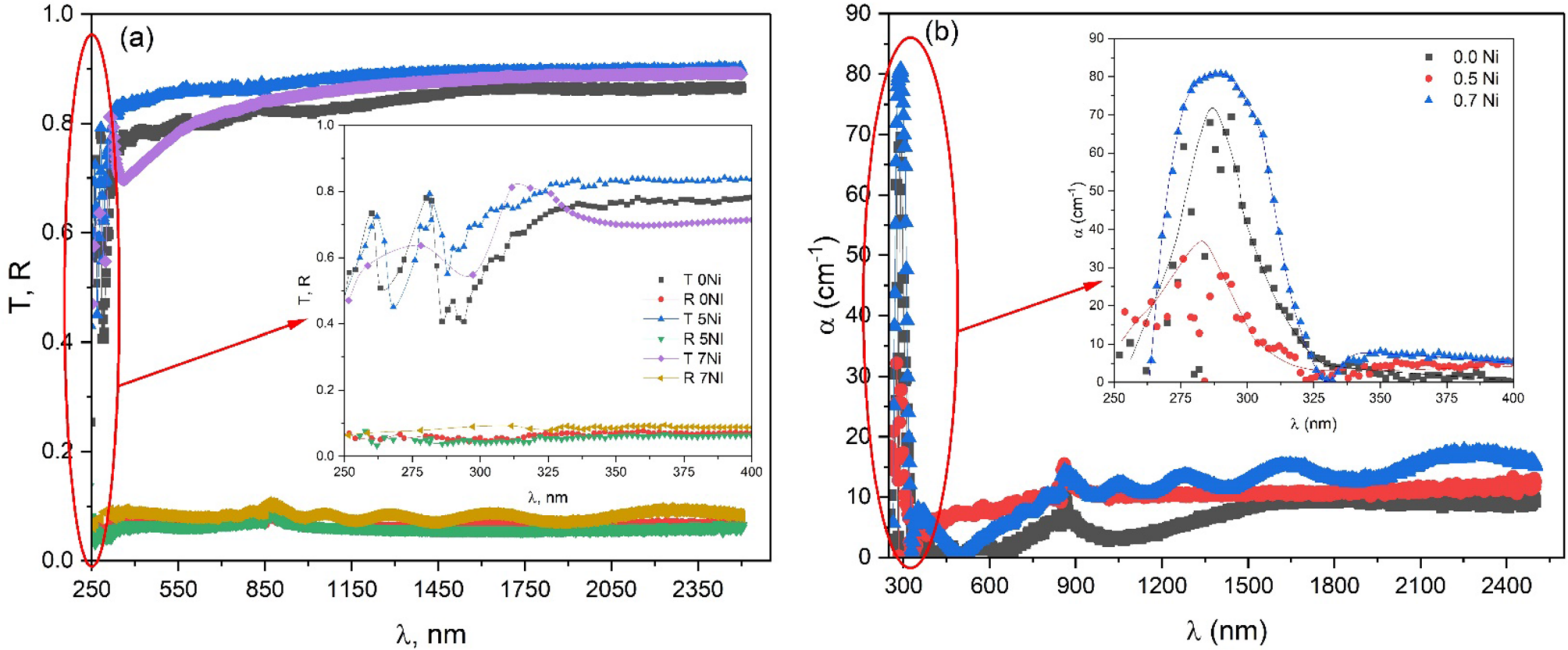
(**a**) The optical transmittance and reflectance spectra, and (**b**) the absorption coefficient of tested films as a function of wavelength.

The optical constants and related parameters of (SiO_2_/MgO/ZrO_2/_CuO) doped with 0, 0.5, and 0.7 Ni^2+^ films have been investigated using UV–Vis–NIR transmittance and reflectance spectrum in the wavelength region of 300–2500 nm. Figure 4a shows the optical transmittance and reflectance spectra of tested films as a function of wavelength.

The general observation shows that the films have strong absorption at short wavelengths and show a transmittance edge at the higher wavelength and these observations indicate the transition of an electron from the valance band to the conduction band. The transmittance edge slightly moves towards a higher wavelength by increasing Ni dopant which can be due to the improvement in crystallinity. Above the transmittance edges, the absorption is negligible and the films become nearly optically transparent. Besides, at low wavelengths, the reflectance of the films shows a sudden decrease and then it reaches a steady state. Moreover, there is a dependence on the Ni content where the transmittance and the corresponding reflectance of the films slightly increase with increasing Ni content which can be due to crystallinity improvement as noted from XRD. With the increase in Ni^2+^ containing through the silicate-based nanoceramics, the surrounding system is a complex matrix from MgO/ZrO_2_/CuO/NiO within the SiO_2_ matrix, which supports a high-crystallinity magnesium copper silicate phase as observed from XRD.

The absorption coefficient (*α*) is an important physical parameter that is frequently used to characterize the light wave’s penetration inside film layers.

The absorption coefficient of the films was calculated with the following formula:

5 $$\alpha d=\mathit{ln}\left[\frac{{\left(1-R\right)}^{2}}{2T}+\sqrt{\frac{{\left(1-R\right)}^{4}}{4{T}^{2}}+{R}^{2}}\right],$$

where the film thickness is d.

The absorption coefficient behavior of the films with different Ni content (0, 5, and 7 wt%) is explored in Fig. 4b. The figure indicates that the films have an absorption peak at about 290 nm wavelengths. At higher wavelengths, the absorption is negligible and saturated. Also, the increasing Ni content leads to an increase in the absorption coefficient that may be due to the increase in light trapping ^44,45^.

**Figure 5:**
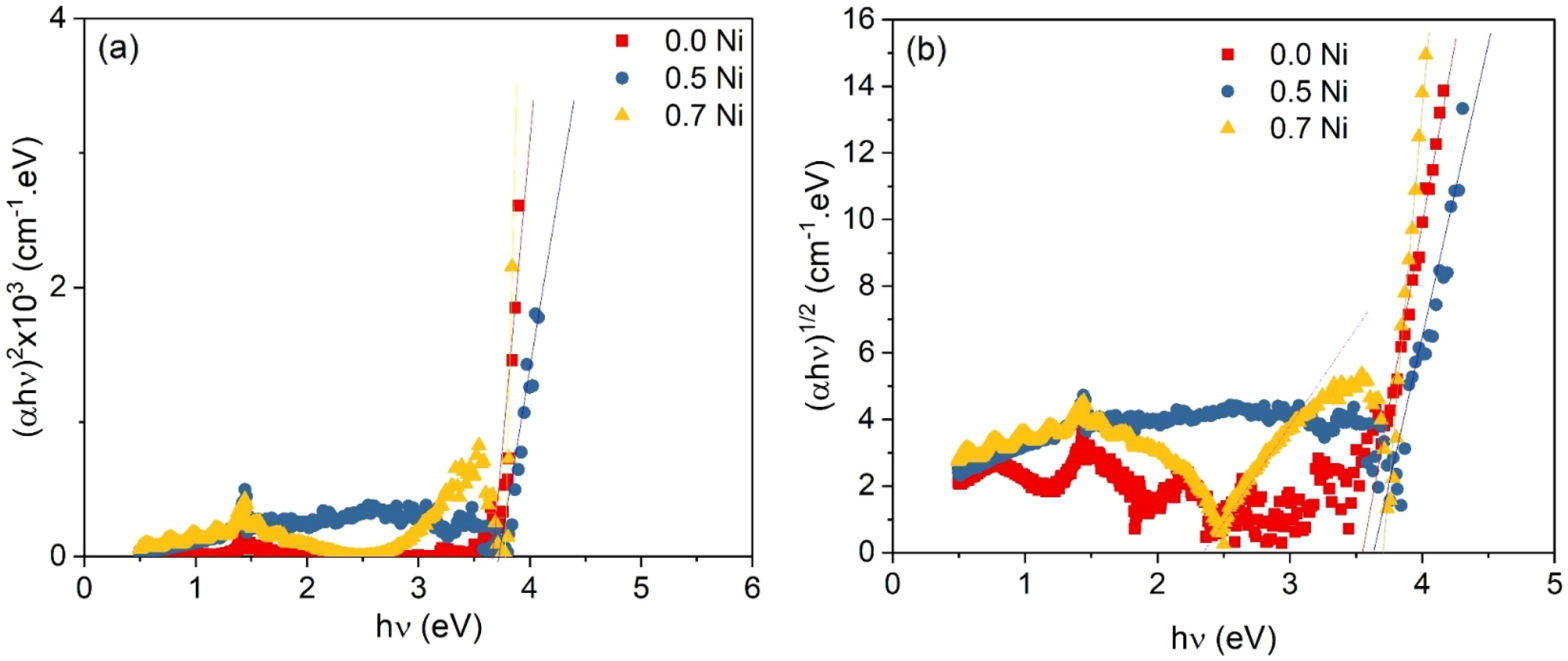
The relation between (αhν)^2^, (αhν)^1/2^, and hν respectively for the films of different Ni content.

The optical transition type and its value can be extracted from the absorption coefficient dependence on the incident light energy through the use of Tauc’s expression ^46^:

6 $$\frac{\alpha h\nu }{p}={\left(h\nu -{E}_{g}\right)}^{r},$$

where p, hν, E_g_, and r represent a transition probability constant, photon energy, optical band gap energy, and an exponent, respectively. The exponent r can take the values of 2, 3, 0.5, and 1.5 which are corresponding to the indirect allowed, indirect forbidden, direct allowed, and directly forbidden transitions, respectively. Figure 5a,b represents the relation between (αhν)^2^, (αhν)^1/2^_,_ and hν respectively for the films of different Ni content. The results in the figure obtain that the direct and indirect transition can take place in that films. The energy of direct transitions of SMZC: Ni (0, 5, and 7 wt%) have the values of 3.66, 3.70, and 3.75 eV, respectively, and that of indirect type are 3.54, 3.62, and 3.71 eV, respectively. Both the direct and indirect values of gap energy slightly increase with Ni content increase and the direct type values are higher than that of indirect type and this is a result of photon, electron, and phonon interaction that is included in the indirect transition. Thus, the indirect transition is more possible than the other type where smaller energy is needed ^47^.

The refractive index (n) is calculated with the next formula ^21^:

7 $$n=\frac{1+R}{1-R}+{\left(\frac{4R}{{\left(1-R\right)}^{2}}-{k}^{2}\right)}^{1/2},$$

where k = αλ/4π, the absorption index, and λ is the light wavelength.

**Figure 6:**
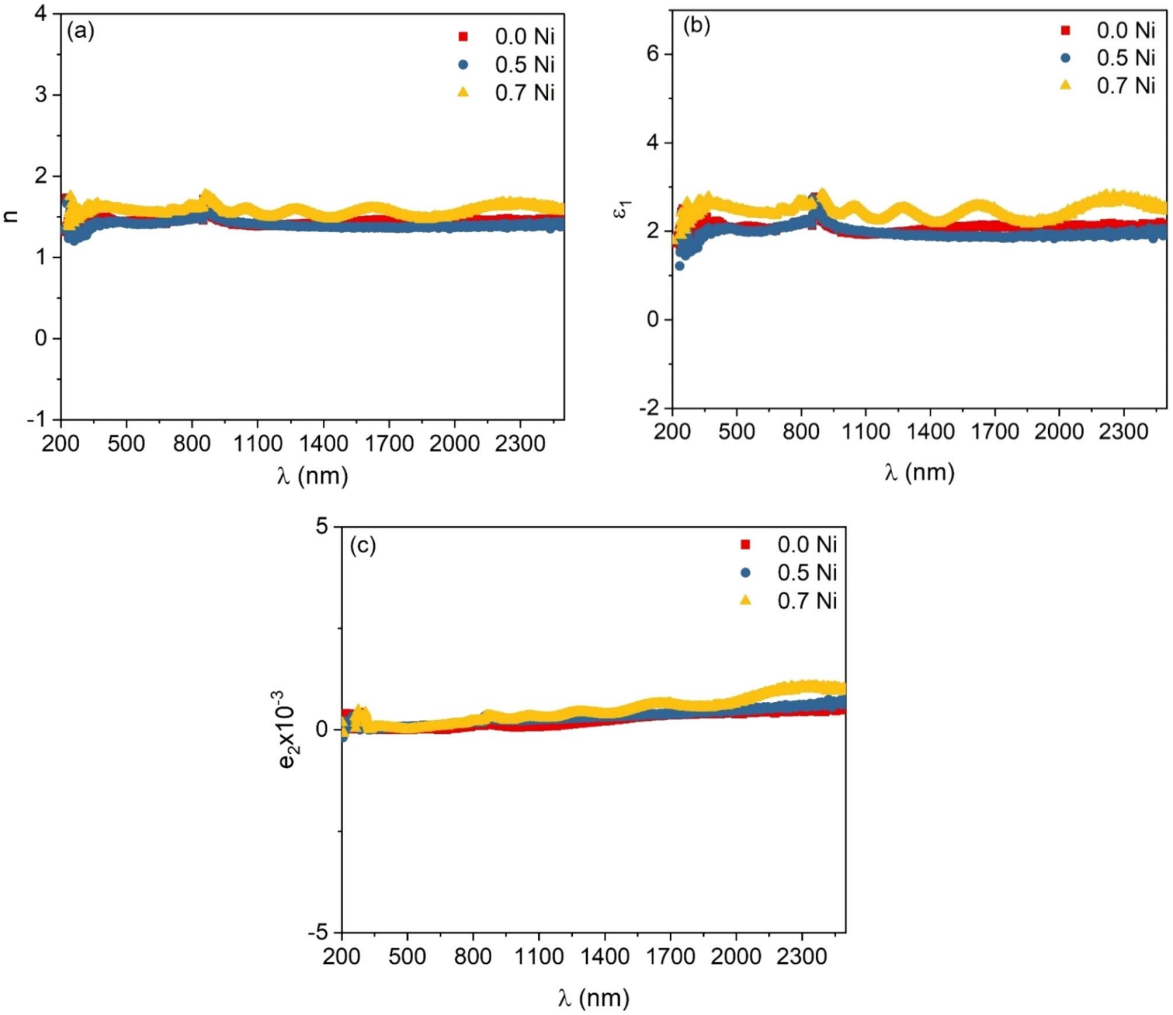
(**a**) The refractive index change, and (**b,c**) the variation of two components of dielectric constant with the photon wavelength of SMZC: (0, 0.5, 0.7)Ni films.

The refractive index change with the wavelength of SMZC: (0, 0.5, 0.7) Ni films are represented in Fig. 6a. from the figure, it is obvious that the values of n are constant with increasing wavelength. It is found that the refractive index of SMZC: (0, 0.5, 0.7)Ni film slightly increases with the increase of Ni content which is referred to as the crystallinity enhancement with the increases of Ni content.

The dielectric real and imaginary parts of the SMZC: (0, 0.5, 0.7)Ni films are extracted by the next formulas:

8 $${\varepsilon }_{1}= {n}^{2}-{k}^{2} \,\mathrm{and} \,{\varepsilon }_{2}=2 n k,$$

where ε_1_ and ε_2_ represent the real and imaginary components of the dielectric constant, respectively.

The variation of two components of dielectric constant with the photon wavelength of SMZC: (0, 0.5, 0.7)Ni films are represented in Fig. 6b,c, respectively. The figure shows that the real component values are higher than that of the imaginary component. The value of ε_1_ shows a constant behavior with increasing the wavelength. As the Ni content increases the real part of the dielectric constant is slightly increased. On the other hand, the imaginary component of dielectric, *ε*_2_, slowly increases with the increase of incident wavelength for all films. Also, *ε*_2_ increases slightly with an increase in Ni content.

### VSM

**Figure 7:**
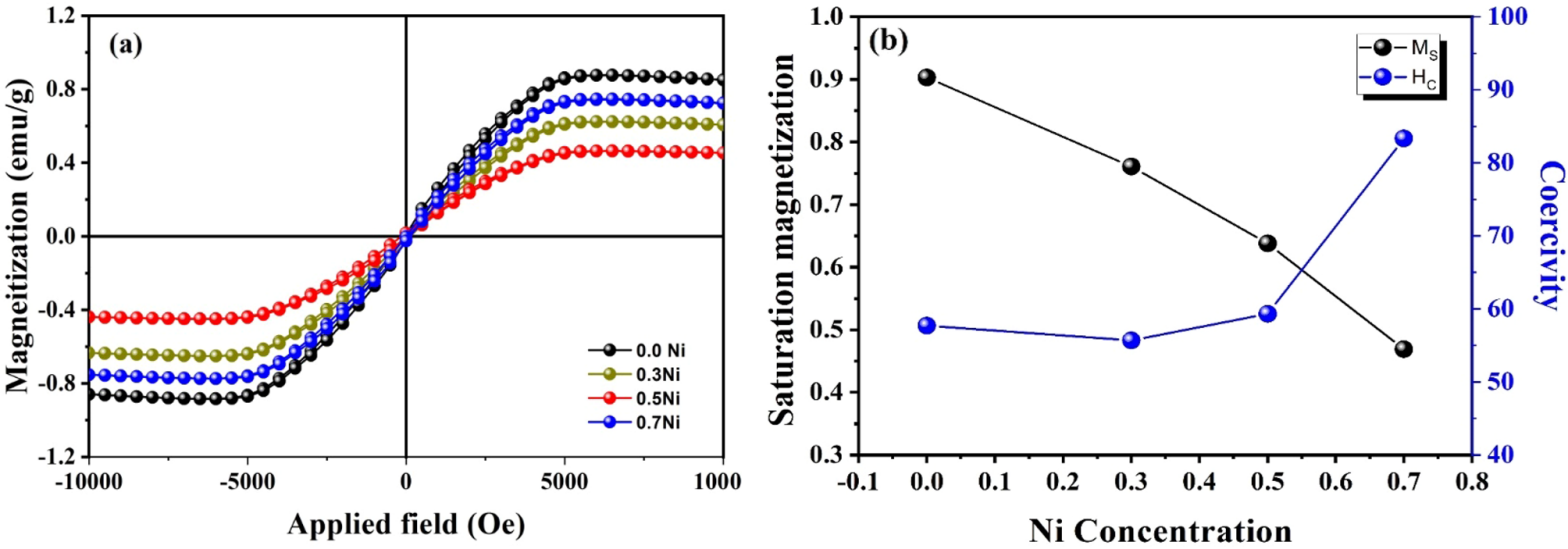
(**a**) The magnetic hysteresis loops of the prepared SMZC: (0, 0.5, 0.7)Ni^2+^nanoceramics within the field sweeping of ± 10 kOe, and (**b**) the propagation of the saturation magnetization and coercivity in terms of the Ni concentration.

The magnetic performance of the prepared SMZC: (0.3, 0.5, 0.7) Ni^2+^ nanoceramics was investigated via a vibrating sample magnetometer within the cycled external field of ± 10 kOe, as the resultant magnetic moment with the applied magnetic field. Most of the magnetic specifications are linked with the magnetic domain state and the particle size and shape ^48^. The behavior of the produced composites resembles the superparamagnetic material attitude with very tiny remanent and negligible coercivity magnitudes. This superparamagnetic behavior is reported with close values of coercivity earlier ^48^. Key magnetic parameters such as (*H*c), (*M*_S_), (*M*_r_), and SQ, extracted from the *M-H* loops (Fig. 7a) are illustrated in Table 2.

**Table 2:** The estimated parameters for the prepared composites.

Sample	*M*_S_ (emu/g)	*Hc (right)* (Oe)	*Hc(left)* (Oe)	*H*_C_ (Oe)	*H*_K_ (emu.Oe/g)	*K (Erg/g)*		*N (Oe*^*2*^* g/Erg)*		n_B_/f.u (µ_B_)
						Equation (11)	Equation (12)	Equations (11 and 17)	Equations (12 and 17)	
0 Ni	0.903	74.12	− 41.29	57.70	52.10	23.52	24.50	− 75.43	− 67.50	0.87
0.3 Ni	0.761	119.49	8.16	55.67	42.36	16.12	16.79	− 96.73	− 94.41	0.71
0.5Ni	0.638	133.86	15.17	59.34	37.86	12.08	12.15	− 134.43	− 133.32	0.59
0.7Ni	0.469	24.88	− 141.83	83.35	39.09	9.17	9.54	− 286.89	− 283.52	0.43

The magnetization at its saturation optimum is determined by the upper limit of the magnetization curve, where the coercivity is calculated from the equation

9 $${H}_{C}=\frac{1}{2}\left[{H}_{c}\left(right\right)-{H}_{c}\left(left\right)\right],$$

where H_c_ right and H_c_ left are the intersection points of the hysteresis loops with the x-axis. A noticeable slight reduction in the saturation magnetization magnitude is remarked in samples by increasing the Ni^2+^ concentration (Fig. 7b), which affirms the successful grafting of nonmagnetic particles on the Ni^2+^ nanoceramics. Other reasons for this reduction might be attributed to the formation of an inactive magnetic layer on the surface of the nanoceramics, or the high dispersion of small particles with an increased magnetocrystalline anisotropy ^49,50^. Or the opposing surface spin antiferromagnetic particles ^51^.

The effective anisotropy constant (K) is expressed by the coercivity as ^52^:

10 $${H}_{K}=\left(\frac{ {H}_{C}\times {M}_{S}}{0.96}\right),$$

or by the anisotropy field (H_K_) as ^53^,

11 $${H}_{K}=\frac{2K}{{M}_{S}}.$$

Thus, a new equation can be deduced for the estimation of the effective anisotropy constant (K) by linking Eqs. (10) and (11) as:

12 $$K=\frac{{H}_{C}\times {M}_{S}^{2}}{1.92}.$$

The saturation magnetization is displayed as the upper limit of the magnetization plot, for the single domain noninteracting nanoceramics a depiction of the relation between the magnetization (*M*) on the y-axis and (1/*H*^2^) on the x-axis as the approximation of Stoner–Wohlfarth theory can give the saturation magnetization (*M*_s_) magnitude as the upper limit of the line intercept with the Y-axis ^54^. This line equation is presented as

13 $$M={M}_{S}\left(1-\frac{B}{{H}^{2}}\right),$$

which can be rewritten in the form of a straight line as

14 $$M=-\frac{{M}_{S}\times B}{{H}^{2}}+{M}_{S}.$$

With the intercept being the saturation magnetization and slope is *M*_S_ × B which gives the constant B value. This value can be inserted into a formula for estimating the effective anisotropy constant as ^54^:

15 $$K=\frac{{M}_{S}\sqrt{15B}}{2}.$$

An inverse proportional is acquired between the coercivity and the crystallite size, This can be expressed mathematically as ^16^:

16 $${H}_{C}=E+\frac{F}{D},$$

where E and F are constants, a plot between the inverse of crystallite size and the coercivity should result in a linear relation.

the demagnetizing factor (N) (i.e. a larger demagnetizing factor is required for higher coercivities) can be obtained using the subsequent formula from Ref.^48^

17 $$N{M}_{S}=\left[\left(\frac{2K}{{\mu }_{0}{M}_{S}}\right)-\left(\frac{{H}_{C}}{0.48}\right)\right],$$

where N is the demagnetizing field which can be calculated by all the pre-estimated other parameters, and µ_0_ is the permeability of the vacuum.

The increase in the coercivity (Fig. 7b) value might be attributed to the elevation of the magnetocrystalline anisotropy (*H*_k_) or the demagnetizing field (N) which requires higher values to overcome the energy barrier. Another possible correlation with the reduction of the crystallite size. The acquired magnetic moment per unit formula in Bohr magneton is expressed by ^52^:

18 $${n}_{B}/\left(f.u.\right)=\frac{molecular \,weight \times saturation\, magnetization}{5585 }.$$

The denominator value results as the product of Avogadro’s no. standard value of 6.022 × 10^23^ mol^−1^, and µ_B_ and the chemical structure of the prepared silicate-based nanoceramics doped without doing Ni^2+^, and 0.3, 0.5, 0.7 doping.

### Effects of studied nanoconjugates interface on tested microbial cell viability

**Figure 8:**
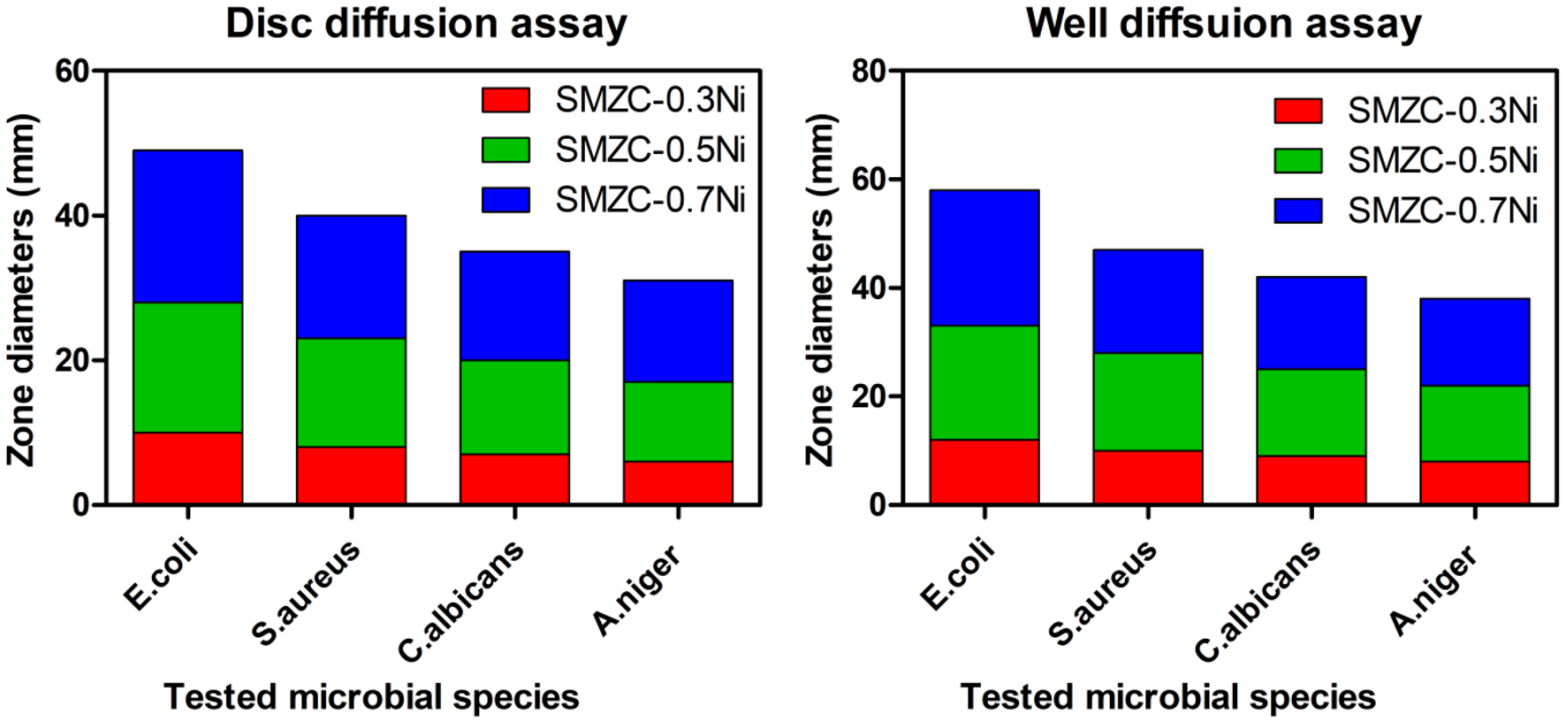
Measured CZD formed from investigated nanocomposites around targeted microbial pathogens.

The antimicrobial effects of the four nanocomposites (SMZC-0.0Ni, SMZC-0.3Ni, SMZC-0.5Ni, and SMZC-0.7Ni) were verified using a qualitative agar diffusion assay and a quantitative total viable cell assay against four species of microorganisms, including (*E. coli* O157:H7*, S. aureus, C. albicans,* and *A. niger)* (Fig. 8). To evaluate the biocidal efficacy of all nanocomposites, the width of the defined clear zone around the discs and the well was determined (Fig. 8). The results showed that SMZC-0.0Ni had no potential biocidal efficacy against all tested microbial species. On the contrary, when compared with SMZC-0.3Ni nanocomposite, simulation results indicated that SMZC-0.5Ni and SMZC-0.7Ni nanocomposites showed significant inhibitory effects against all tested pathogens (*E. coli, S. aureus, C. albicans,* and *A. niger*). Using the well diffusion assay, the researchers discovered that the SMZC-0.7Ni nanocomposite presented a cumulative inhibition zone of 25, 19, 17, and 16 mm versus *E. coli, S. aureus, C. albicans,* and *A. niger*, in contrast.

On the other hand, data represented in Fig. 8, confirmed that SMZC-0.3Ni nanoceramic had the shortest apparent area width compared to the species mentioned above in both disc assays and well diffusion. In addition, according to the results, when compared to the other tested species, *E. coli* O157:H7 was more susceptible to all analyzed nanoceramics. Moreover, compared with different bacterial strains, the fungal species were more resistant to all the nanocomposites, which could be due to the rigid cell walls, which protect them from the lethal effects of the investigated nanocomposites.

### Determination of minimal inhibitory concentration and surviving cells

**Figure 9:**
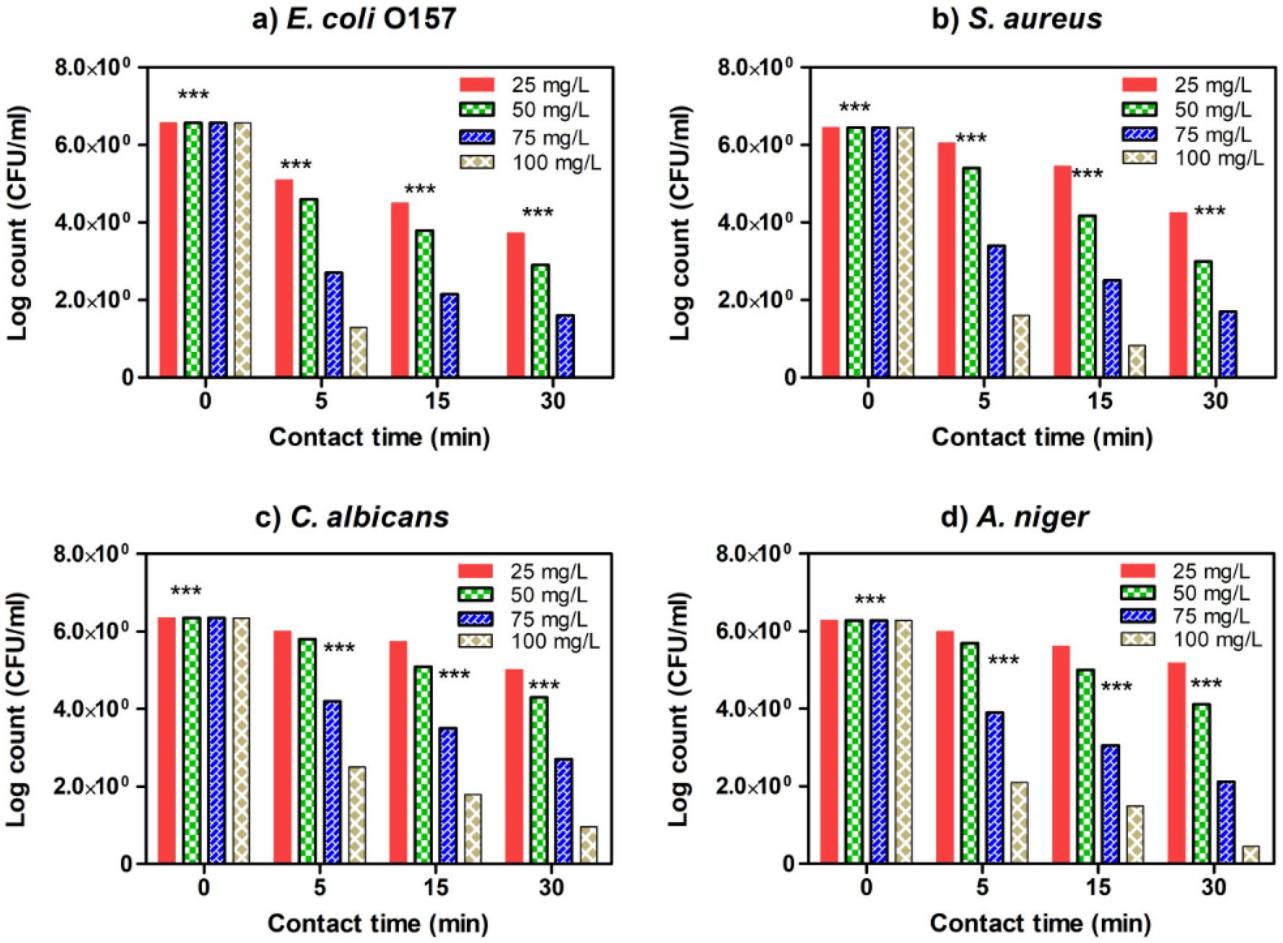
Estimated MIC values of SMZC-0.3Ni towards four tested microbial species, including (**a**) *E. coli*, (**b**) *S. aureus*, (**c**) *C. albicans*, and (**d**) *A. niger*. The remaining viable cell populations at various time intervals of 5, 15, and 30 min are also displayed.

**Figure 10:**
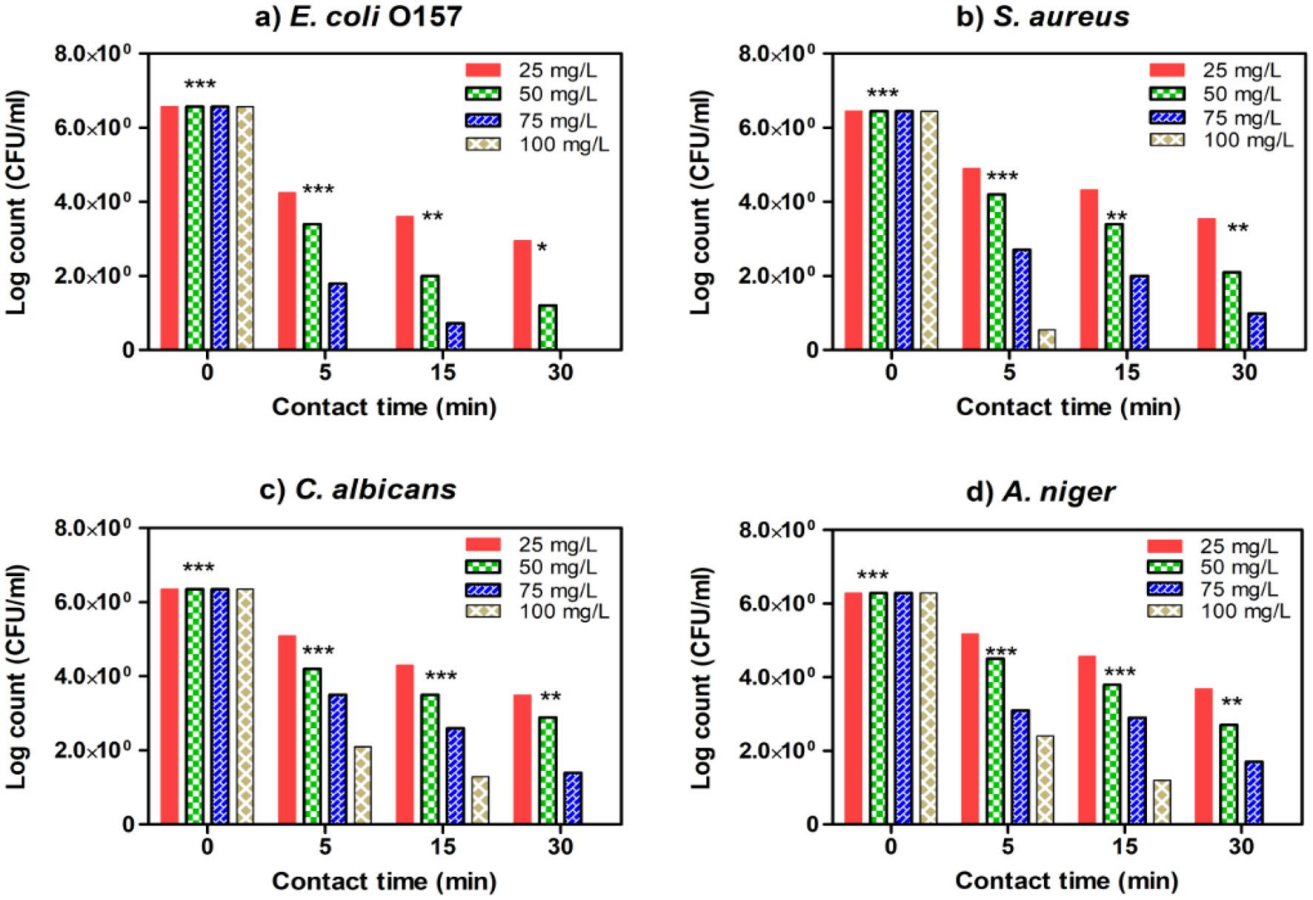
Estimated MIC values of SMZC-0.5Ni towards four tested microbial species, including (**a**) *E. coli*, (**b**) *S. aureus*, (**c**) *C. albicans*, and (**d**) *A. niger*. The remaining viable cell populations at various time intervals of 5, 15, and 30 min are displayed.

**Figure 11:**
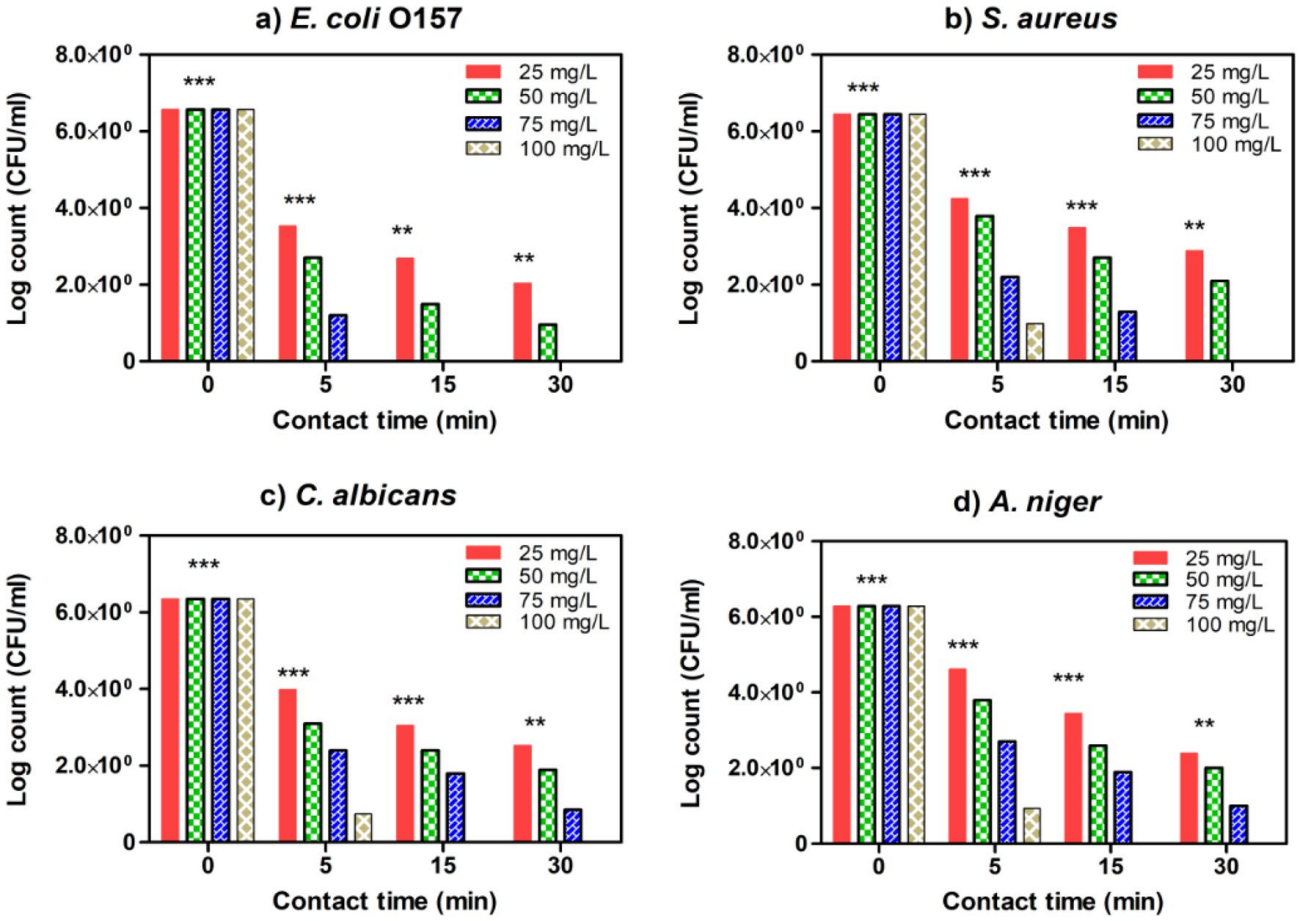
Estimated MIC values of SMZC-0.7Ni towards four tested microbial species, including (**a**) *E. coli*, (**b**) *S. aureus*, (**c**) *C. albicans*, and (**d**) *A. niger*. The remaining viable cell populations at various time intervals of 5, 15, and 30 min are displayed.

The observed results are illustrated graphically in Figs. 9, 10 and 11, the MIC values and inhibitory effect of all studied nanocomposites (SMZC-0.3Ni, SMZC-0.5Ni, and SMZC-0.7Ni). The lethal dose of SMZC-0.3Ni was 75 mg/L in 10 min for *E. coli* and 15 min for *S. aureus*, while data showed that SMZC-0.3Ni could not wholly eliminate fungal species without diminishing log counts (Fig. 9). Results illustrated in Fig. 10 displayed that SMZC-0.5Ni nanocomposite had a more significant deadly dose for the pathogens studied than SMZC-0.3Ni nanocomposite antiseptic). Results obtained exhibited that SMZC-0.7Ni nanocomposite had a superior inhibitory effect and total mortality in overall log counts for all examined species, the levels of the lethal dose of SMZC-0.0Ni towards *E. coli*, *S. aureus* were 50 mg/L within 10 min, and for *S. aureus* and fungal species, 100 mg/L within 10 min for, *C. albicans* and *A. niger* (Fig. 11). Interestingly, SMZC-0.5Ni and SMZC-0.7Ni nanocomposites revealed a decrease in the populations of all examined species. Consequently, SMZC-0.3Ni nanocomposites had a greater antimicrobial action than SMZC-0.3Ni and SMZC-0.5Ni nanocomposites. Gram-negative and positive bacterial species (*E. coli* and *S. aureus*) were both sensitive to all of the nanocomposites examined, but there was no difference between the two molds and yeast strains whenever it came to the SMZC-0.3Ni nanocomposite.

Although the interpretations are in the same line with the findings of El Nahrawy et al. ^10^, who and her co-authors perceived that the Cu-NPs have excellent antimicrobial features. Findings by similar researchers that bacterial species are more susceptible to CuSi NPs than mold and yeast species *(C. albicans* and *A. niger*) approved with the results of this investigation. The combined antibacterial effects of Cu and Si NPs are attributed to the disruption of the pathogen’s cell membrane, expected due to the presence of OH radicles, as it can penetrate the cellular layers, causing severe damage to the cell wall ^8^. The powerful antimicrobial efficacy of ZrO_2_ against *E.coli* was more than 99 percent but was only about 46 percent against *S. aureus* ^55^.

### Estimation of released protein

**Figure 12:**
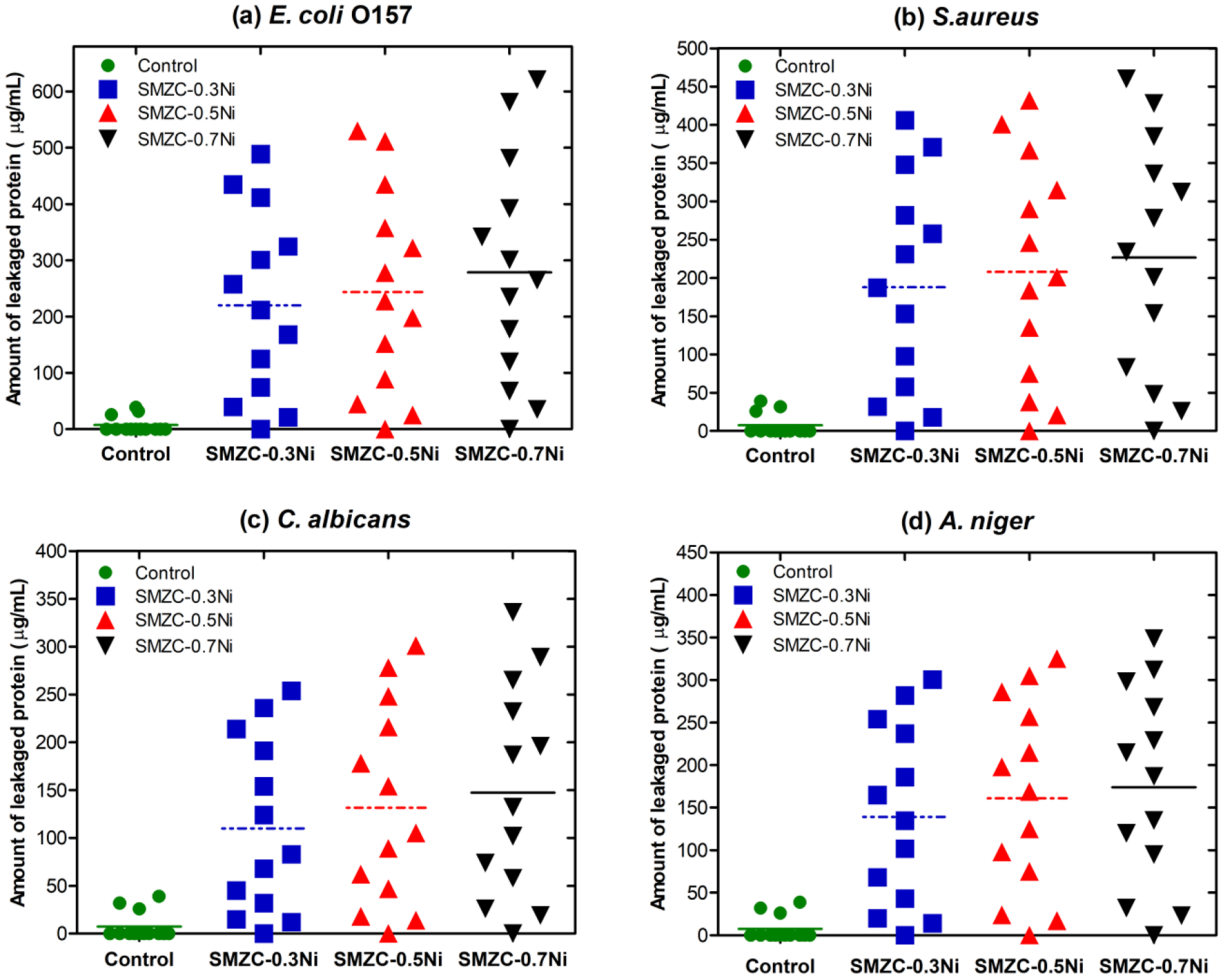
Quantified produced intercellular protein of targeted microbes (**a**) *E. coli*, (**b**) *S. aureus*, (**c**) *C. albicans*, (**d**) *A. niger* after exposure by the effective dose of four investigated nanocomposites.

The results are depicted graphically in Fig. 12, demonstrating that the amounts of liberated protein from targeted species before subjecting to examined nanocomposites were low. However, these amounts of produced protein were increased gradually according to the types of species and examined nanocomposites. It’s worth noting that all of the microbial pathogens examined revealed a considerable amount of protein after receiving the lethal dose of SMZC-0.7Ni nanocomposites was swiftly increased. The estimated protein liberated from *E. coli, S. aureus, C.albicans, and A. niger* were recorded as 621, 460, 336, and 349, respectively. Several investigations revealed that the most considerable amount of protein produced indicates the examined nanocomposites’ effectiveness in destroying the cell wall, releasing cellular contents outside ^10^.

### The kinetic modeling of the killing rate of the tested microbes

**Figure 13:**
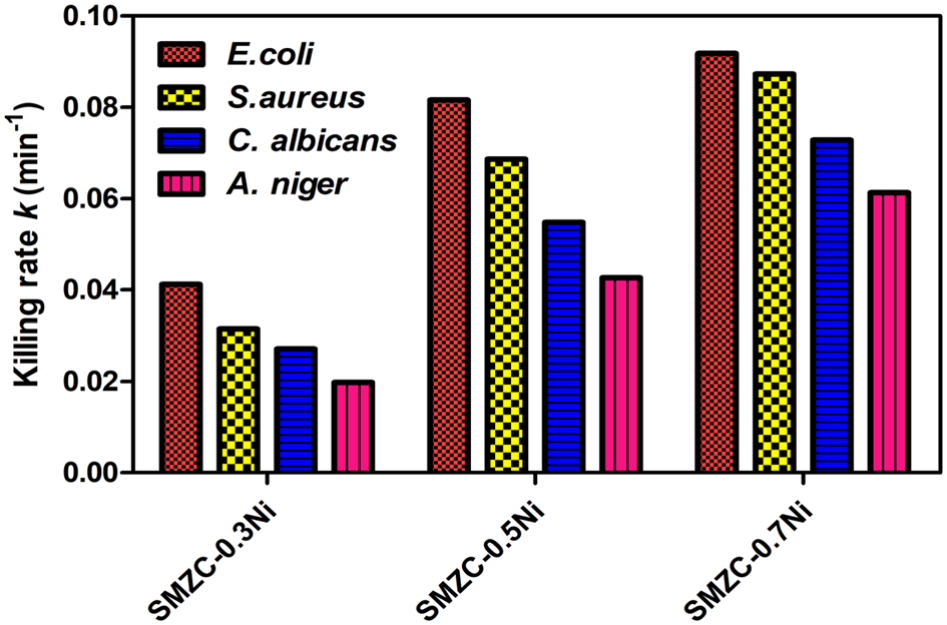
Decaying speed of targeted pathogenic microbes by 100 mg/L of examined nanocomposites via pseudo-first-order kinetic modeling.

The findings of kinetics modeling employing pseudo-first-order were depicted in Fig. 13; it was established that *E. coli* O157:H7 seemed to have the fastest destruction frequency of all constructed nanocomposites, whereas the filamentous fungi would have the shortest frequency. Interestingly, the fabricated SMZC-0.7Ni nanocomposite’s effective dose was the only one that could significantly limit the growth of microorganisms in a faster manner than those of other nanocomposites. It was necessary to keep in mind that the species of *A. niger* investigated were being destroyed over a significant period.

### Toxicity performance testing

Nanocomposites are broadly applied in various bio-applications, but notwithstanding nanobiotechnology’s rapid advancement and early acceptability, the occurrence of negative health implications from prolonged exposure at various concentration ranges in humans and the environment has yet to be determined. The influence on the environment of NPs, on the other hand, is expected to increase in the future. The capacity of NPs to organize around protein content is influenced by particle diameter, curve, form, surface composition, charge, bifunctional moieties, and renewable power ^56^. The average EC50 value of all the tested nanocomposites was 214 for SMZC-0.3Ni, 198 for SMZC-0.3Ni, 208 for SMZC-0.5Ni, and 173 for SMZC-0.7Ni after 30 min of incubation (Table 3).

**Table 3:** The values of effective concentrations EC50 (mg/L) after 5, 15, and 30 min of exposure to examined nanocomposites.

Examined nanocomposites	Exposure time (min)	Results	Standard values	
		EC_50_ conc.	EC_50_% degree	Toxicity level
SMZC-0Ni	After 5	238	0–19	Extremely toxic
	After 15	221	20–39	Very toxic
	After 30	214	40–59	Toxic
SMZC-0.3Ni	After 5	225	60–79	Moderately toxic
	After 15	213	≥ 100	Non-toxic
	After 30	198		
SMZC-0.5Ni	After 5	228		
	After 15	217		
	After 30	208		
SMZC-0.7Ni	After 5	204		
	After 15	185		
	After 30	173		

This is attributed to the fact that the nanocomposites examined are harmless and have no deleterious impacts; consequently, they may be used in various fields, encompassing ecological applications such as water decontamination, food packaging, and the textiles sector. The findings are following El Nahrawy et al. ^57^, who found that copper is non-toxic when mixed with nanoceramic materials. The study also revealed that while Zr NPs are harmful, their toxicity has been reduced in this nanoceramic. As a result of its effectiveness, it could be administered as a powerful antibacterial without even being poisonous or environmentally destructive ^58^.

### Wastewater decontamination using innovative nanocomposites

**Figure 14:**
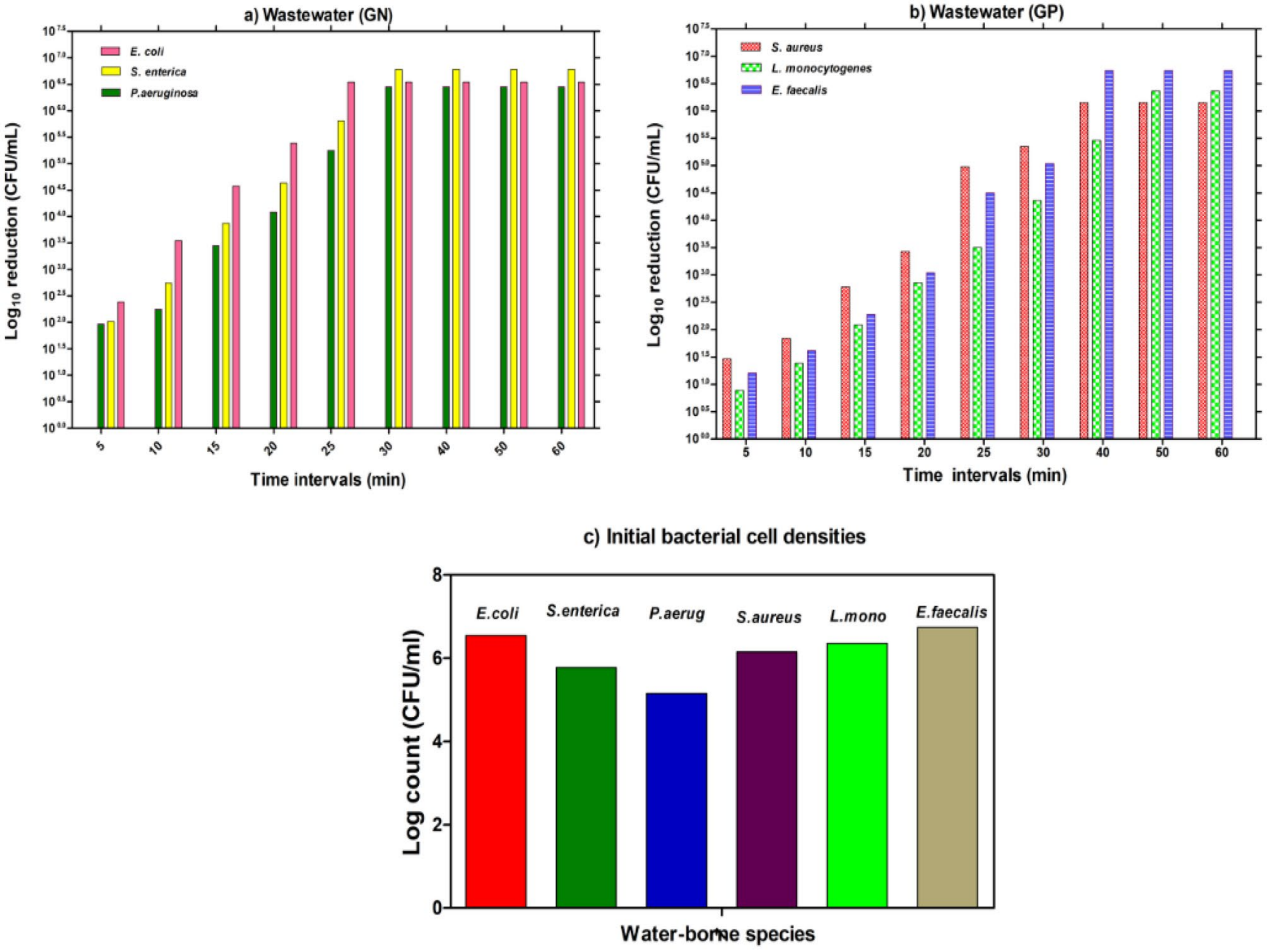
Disinfection of wastewater samples using 100 mg/L of SMZC-0.7Ni during various time intervals.

Based on the above experimental results, it could be considered the SMZC-0.7Ni the best choice to apply as an alternative disinfecting agent for the wastewater disinfection process. Figure 14, illustrates the disinfection efficiency of SMZC-0.7Ni as a promising deactivator for some waterborne pathogens. Results displayed that the effective dose of SMZC-0.7Ni could wholly deactivate all estimated waterborne pathogens. The different interaction time (25 and 30 min) for eliminating the growth of *E. coli, S. enterica,* and *P. aeruginosa* was recorded. While *S. aureus, L. monocytogenes, and E. faecalis* required a prolonged interaction time (40 and 50 min) to be completely inactivated. Likewise, Xu et al. ^59^ revealed that 99% of log 5 of the bacterial populations of some respective waterborne pathogens such as *E. coli, Pseudomonas aeruginosa, Acinetobacter*, and *Bacillus* have been reduced after disinfecting with some nanocomposites.

## Conclusion

High-crystallinity Ni^2+^ doped silicate-based nanoceramics have been grown utilizing a controlled sol–gel process and calcined at 800 °C. XPS, XRD, and TEM analyses reveal that the SMZC and doped nanoceramics consist of a crystalline SMZC matrix containing Ni^2+^ in the crystalline structures. Optical analysis shows that the samples except both direct and indirect transition with values of 3.66, 3.70, and 3.75 eV (direct case), and 3.54, 3.62, and 3.71 eV (indirect case), for SMZC: (0, 0.5, and 0.7)Ni^+2^, respectively. The refractive index is constant with increasing wavelength and slightly increases with increasing Ni content. The magnetic results reveal that the existence of a higher magnetization depends on the SMZC compositions, as they exhibited a smooth hysteresis loop, with a slight reduction in their saturation magnetization magnitude with increasing the Ni^2+^ concentration. The antimicrobial activities results exhibited that SMZC-0.7Ni has outstanding antimicrobial properties, particularly against the investigated positive and negative bacterial species, as well as fungal strains. Consequently, the SMZC-0.7Ni nanocomposites exhibit exceptional inhibitory properties and may be applied for decontaminating wastewater by eradicating all harmful waterborne microorganisms.

## Data Availability

The datasets used and/or analyzed during the current study are available from the corresponding author on reasonable request.
